# Evaluation of the Porosity and Morphology of Microstructured Charcoal

**DOI:** 10.3390/ma18081730

**Published:** 2025-04-10

**Authors:** Andrzej Biessikirski, Michał Dworzak, Grzegorz Piotr Kaczmarczyk, Grzegorz Machowski, Magdalena Ziąbka, Agata Kaczmarczyk, Joanna Jakóbczyk, Suzana Gotovac-Atlagić

**Affiliations:** 1 Faculty of Civil Engineering and Resource Management, AGH University of Krakow, 30-059 Krakow, Poland; dworzak@agh.edu.pl (M.D.); jjakob@agh.edu.pl (J.J.); 2 Faculty of Geology, Geophysics, and Environmental Protection, AGH University of Krakow, 30-059 Krakow, Poland; machog@agh.edu.pl; 3 Faculty of Materials Science and Ceramics, Department of Ceramics and Refractories, AGH University of Krakow, 30-059 Krakow, Poland; ziabka@agh.edu.pl; 4 Independent Researcher, 30-059 Krakow, Poland; agata_kaczmarczyk@icloud.com; 5 Faculty of Natural Sciences and Mathematics, University of Banja Luka, Mladena Stojanovića 2, 78000 Banja Luka, Bosnia and Herzegovina; suzana.gotovac.atlagic@unibl.org

**Keywords:** charcoal, SEM, mercury intrusion porosity, tomography, wall thickness, fibrous structures

## Abstract

This study presents a comprehensive assessment of the morphology and porosity of microstructured charcoal using a combination of scanning electron microscopy (SEM), computed tomography (CT), and mercury intrusion porosimetry (MIP) methods. SEM analysis revealed a parallel arrangement of tube-like structures interspersed with smaller pores, confirming the presence of fibrous formations. MIP evaluation was conducted in two research series. MIP results identified macropores as the primary contributors to mercury intrusion; however, a minor volume of mercury also intrudes to the mesopores. The total pore area was determined to range between 70.7 and 88.5 m^2^·g^−1^, with porosity values of approximately 58.0–62.4% across different experimental series. These variations highlight the heterogeneous nature of the sample. Additionally, the uniformity of the charring process during dry wood distillation was indicated by wall thickness measurements, which ranged narrowly from 5.7 to 25 µm.

## 1. Introduction

Carbon-based materials have broad industrial applications, largely due to their sorption properties [1,2,3]. Among these, activated carbon stands out as one of the most widely employed sorbents, owing to its highly developed microporous structure and significant surface area, which enhance its adsorption capacity [4,5,6]. Additionally, emerging carbon allotropes, including nanotubes and fullerenes, have been investigated for their potential as advanced sorption materials [7,8,9,10,11,12]. Charcoal is another promising carbon-based material, favored for its simple production process and resource availability. However, its application as a sorbent is often limited by low porosity. This is attributed to the presence of resins and incomplete combustion products that fill the pores, reducing its absorbability [13]. Enhancing porosity through chemical or gas activation can produce activated charcoal, but this involves energy-intensive processes that may diminish its economic feasibility for industrial-scale use.

Existing research has predominantly focused on understanding the porosity and surface morphology of charcoal derived from different feedstocks under varying synthesis conditions. For instance, Abe et al. explored pore volume and macropore size distributions in charcoal from various wood types [14]. Costa et al. examined the chemical, hygroscopic, and morphological properties of eucalypt-derived charcoal carbonized at temperatures ranging from 340 °C to 460 °C [15]. Somerville and Johanshahi studied the effects of pyrolysis temperature and compression on the porosity and density of eucalypt charcoal [16]. Similarly, Ramos et al. investigated short-rotation forestry eucalypt species with respect to carbonized wood porosity and morphology [17]. High-temperature pyrolysis of spruce and oak charcoal has been extensively studied by Surup et al., who analyzed nanostructures via TEM and porosity using mercury intrusion porosimetry (MIP), SSA, and DFT methods across pyrolysis conditions of 800 °C, 1200 °C, and 1600 °C [18]. They indicated that the pore surface of charcoal samples was in the range between 11 and 69 m^2^·g^−1^ depending on the wood and process condition. Dufourny et al. conducted comparable studies on spruce and eucalypt wood, assessing its potential as a reducing agent [19]. Hared et al. investigated the development of porosity in wood impregnated with phosphoric acid during pyrolysis for activated carbon production, identifying optimal conditions to maximize the specific surface area [20]. Based on this, it can be stated that a limited number of research works presented information either on the surface area or porosity of charcoal. On the other hand, there were some research papers in which coals were subjected to porosity evaluation. The most important is Friesen and Mikula’s study, where they shows that lower-rank Canadian coals are characterized depending on the type by the surface areas of 14–50 m^2^·g^−1^ [21].

Further research includes surface and structural analyses, such as those conducted by Fraga et al. on tar-impregnated charcoal for cokemaking [22], Kuang et al. on bamboo charcoal embedded with SiO_x_ [23], and Mariana et al. on candlenut shell charcoal [24]. Additionally, Pastor-Villegas et al. examined the pore structure of holm oak and eucalyptus chars heated between 250 °C and 1000 °C [25], while Rampe et al. studied pore length in coconut shell charcoal pyrolyzed at 300 °C and activated with HCl [26]. Milani et al. analyzed the structure of charcoal fibers from banana species [27], and Huang et al. assessed the impact of carbonization temperature on microporosity in Chinese firewood charcoal [28]. These studies emphasize the significant focus on optimizing charcoal porosity to enhance its sorption characteristics. To maximize functional performance, sorbents should ideally be derived from widely available materials using one-step, energy-efficient processes, while maximizing porosity.

This research presents a systematic analysis of the porosity and morphology of a unique charcoal sample containing fibrous carbon structures in the form of nuggets. This novelty related to fibrous structures in the charcoal sample requires us to conduct a more detailed examination of the porosity, not only limited to the results of a N_2_ isotherm adsorption previously presented by us in [29]. It should be taken under consideration that the adsorption isotherms often underestimate surface area compared to MIP, especially in the case of microporous structures, which are present in the tested sample. This work aims to contribute to the understanding of sample structural properties, enabling advancements in the applicability of charcoal in NO_x_ filter systems.

## 2. Materials and Methods

### 2.1. Materials

The microstructured charcoal (MC) sample was synthetized at the HI Destilacija Teslić, Bosnia and Herzegovina plant by a dry wood distillation process.

The process begins with the natural and artificial drying of Bosnia and Herzegovina’s beech trees, utilizing hot air produced in caloric furnaces and heat derived from burnt gases generated within the system. The next stage involves the charring of wood. Once transported to the furnace, the furnace door is securely closed to prevent air infiltration. Heating pipes located at the base of the furnace provide the required thermal energy, while a dedicated opening allows the collection of steam and gases, channeling them into a central pipe shared by multiple furnaces. The charring process unfolds in distinct stages. The initial stage occurs at temperatures between 150 °C and 180 °C, during which free water is eliminated from the wood. The second phase, taking place at temperatures of 180 °C to 260 °C, is characterized by the formation of gases such as carbon dioxide (CO_2_), carbon monoxide (CO), acetic acid vapor, methanol, and tar. The final phase of carbonization occurs at temperatures ranging from 270 °C to 360 °C, during which a transition in gas composition is observed. In this stage, the concentrations of CO_2_ and CO decrease, while methane, hydrogen, ethylene, and acetylene are formed. This phase also witnesses intense production of acetic acid, methanol, and tar. Upon completion of the charring process, the charcoal is cooled in a water basin. Residual gases and vapors are subsequently collected and directed to a condenser, where condensation yields a raw wood vinegar containing tar.

The MC particles exhibit irregular shapes resembling nuggets, with diameters ranging from 1 to 2 mm. Detailed Raman spectroscopy, X-ray diffraction (XRD), and infrared (IR) spectroscopy analyses of the pure MC sample have been previously reported in [29].

### 2.2. Methods

Scanning electron microscopy (SEM) analysis was conducted using a Nova NanoSEM 200 (FEI, Eindhoven, The Netherlands). The acquired micrographs were obtained at magnifications ranging from 350 to 10,000×. Observations were performed under a low-vacuum environment (approximately 60 Pa) using a low vacuum detector (LVD) operating in secondary electron mode. The electron beam was set to an accelerating voltage of 15 kV.

A computer tomographic analysis (CT) was conducted using a GE Phoenix v|tome|x M instrument (General Electric Company, Hürth, Germany), equipped with a microfocus X-ray tube. To ensure stability during the scan, the sample was affixed to a low-absorption foam mount using an adhesive. While the adhesive was visible in the reconstructed cross-sectional images, it was selected to minimize interference with the imaging process.

The scanning parameters included a tube voltage of 30 kV and a tube current of 550 µA, producing a voxel resolution of 5.53 µm^3^, which allowed for detailed visualization of the charcoal’s microstructure. A total of 3000 X-ray projections were acquired, ensuring comprehensive data coverage for volumetric reconstruction.

The acquired projection data were processed and reconstructed into a three-dimensional dataset using GE datos|x software (version 2.7.2 RTM 2019). Advanced corrections were applied during reconstruction to enhance image quality: Adaptive Gradient Correction (AGC) filter was used to reduced noise and improve the clarity of the reconstructed slices, geometry calibration was applied to minimized spatial distortions by ensuring accurate alignment of the scanning components, and Beam-Hardening Correction (BHC+) was used to address beam-hardening artifacts, particularly for the heterogeneous coal matrix, using a power setting of 6.3.

These processing techniques ensured high-resolution imagery with minimal artifacts while preserving the coal sample’s structural details. The dataset provided insights into critical features such as porosity and wall thickness. Although the adhesive was present in some images, it did not significantly affect the integrity of the coal matrix analysis.

Wall thickness analysis, essential in materials science and quality control, was conducted using VGStudio Max 3.5.1 software, employing the Sphere Method. This method offers precise and efficient evaluation of complex geometries derived from CT scan data. The Sphere Simulation allowed the algorithm to simulate spheres expanding within the 3D geometry. For each surface point, the largest possible sphere fully enclosed within the object is identified, representing the local thickness at that point; an Inner-Outer Surface Mapping method determines the radius of spheres that fit entirely between the object’s inner and outer surfaces, and Color-Coded Visualization means that the thickness data is visualized as a gradient map overlaid on the 3D model, with color variations highlighting regions of uniformity or critical deviations. This approach facilitated detailed wall thickness measurements of the coal sample, enabling precise characterization of structural uniformity and deviations. Detailed description of the CT method, wall thickness method, and general process flowchart was provided in [30].

Mercury Intrusion Porosimetry (MIP) was made in AutoPore IV 9520 produced by Micrometrics (Atlanta, GA, USA). Prior to measurements, samples were dried at 110 °C for 24 h. This method requires the application of pressure to infiltrate the material’s pores. Pore diameter is calculated using the Washburn. A detailed description of the method was presented in [30].

## 3. Results and Discussion

**Figure 1:**
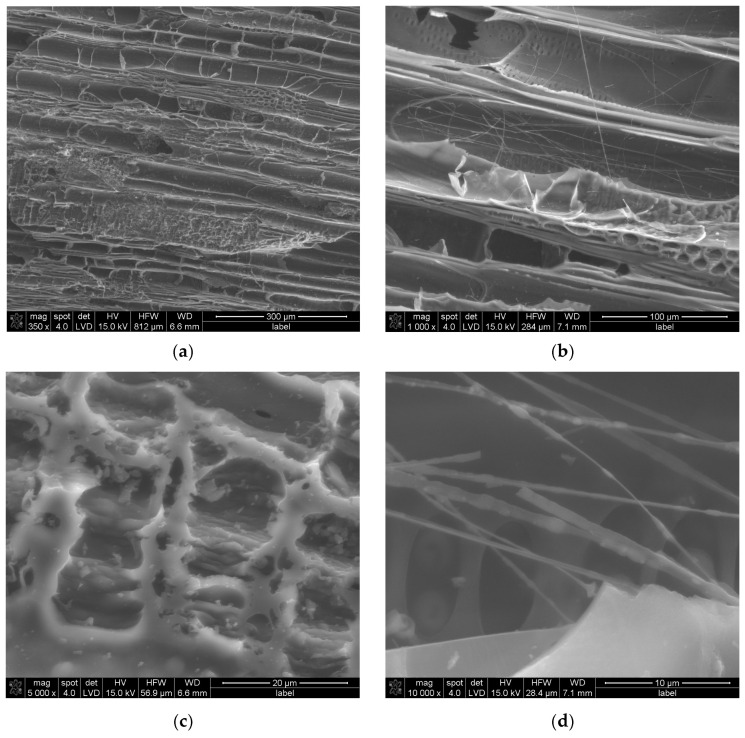
SEM results of MC sample at magnifications of (**a**) 350×, (**b**) 1000×, (**c**) 5000×, and (**d**) 10,000×.

SEM investigation, Figure 1, shows that under magnification of 350×, the research sample reveals parallel structures resembling half-cylinder tubes distributed across the entire surface. Additional smaller and deeper pores are observed, such as those visible in the upper-right corner of Figure 1a. A higher magnification of 1000×, Figure 1b, highlights structural deformations, including grooves located along the tube-like structures, consistent with findings reported in our previous work [29]. Irregularities are apparent between the tubes and along their edges, akin to observations made by Elsaid et al. [31] near porous structures. At random locations, fibrous, elongated structures are discernible. When magnified to 5000×, Figure 1c, macro- and mesopores become evident. These pores are not confined to specific complexes, as seen in Figure 1a, but are also present across various surfaces of the material. Within these pores, smaller, deeper voids are apparent, corroborating findings by Elsaid et al. [31]. Jjagwe et al. attribute the formation of extended pores to a high ligno-cellulosic content [32]. Further magnification at 10,000× reveals randomly oriented fibrous structures, some appearing entangled, as in Figure 1d. These fibrous formations likely enhance the surface area of the material, which could positively influence its adsorption properties. This structural complexity suggests potential applications in areas requiring high adsorption efficiency. Further evaluation of the pore size distribution, wall thickness, and grain morphology was carried out by CT.

**Figure 2:**
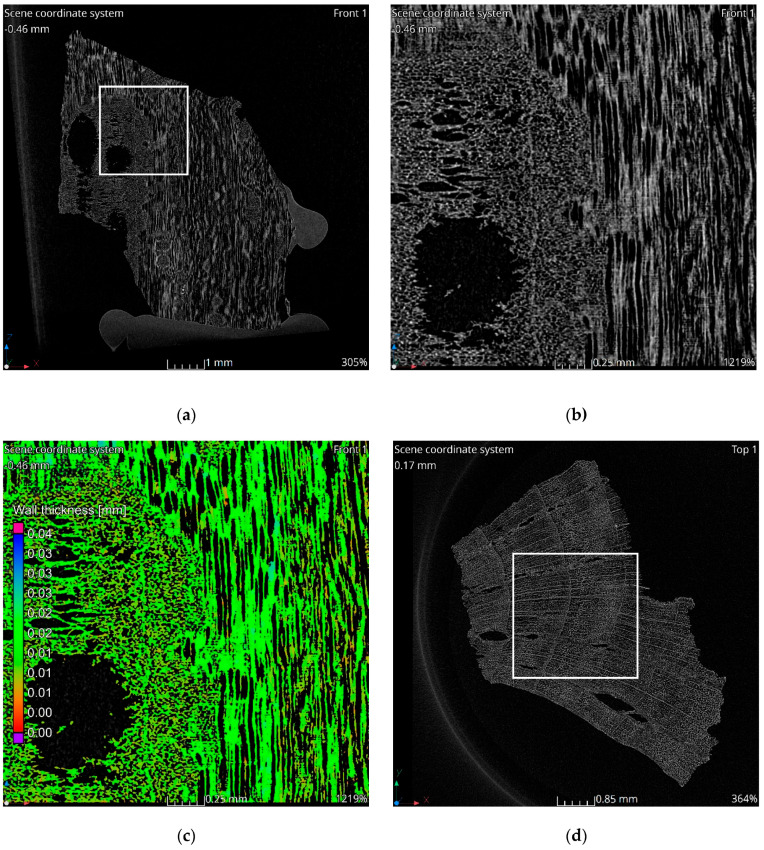

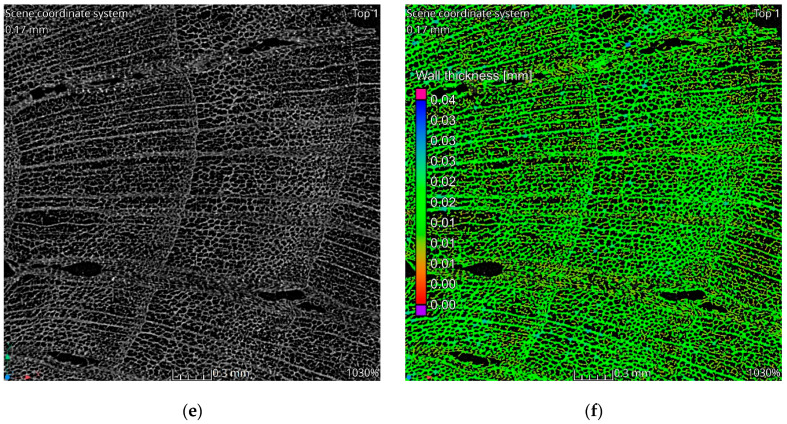
Results of tomographic analysis: (**a**) longitudinal section; (**b**) longitudinal section zoom; (**c**) longitudinal section visualization of wall thickness analysis results; (**d**) cross-section; (**e**) cross-section zoom; (**f**) cross-section visualization of wall thickness analysis results.

The tomographic data reveal a structure consistent with the natural morphology of wood. Tomographic images, Figure 2a–c, illustrate the alignment along wood fibers, while the images in Figure 2d–f display cross-sections perpendicular to the fibers. The observed structure exhibits significant heterogeneity. In the cross-sectional images, two distinct regions are evident. The left side of Figure 2c corresponds to a knot area within the wood structure, characterized by larger pores and channels. Qualitative analysis indicates that the material walls in this region are notably thinner compared to the surrounding areas. This observation is corroborated by the wall thickness analysis visualization, which highlights a higher proportion of red-colored voxels in the knot region, indicative of reduced wall thickness, Figure 2c,f. Seasonal growth rings corresponding to summer and winter wood are discernible in the cross-sectional images. Regions associated with thinner walls align with areas containing larger pores, reflecting the growth ring variations.

**Figure 3:**
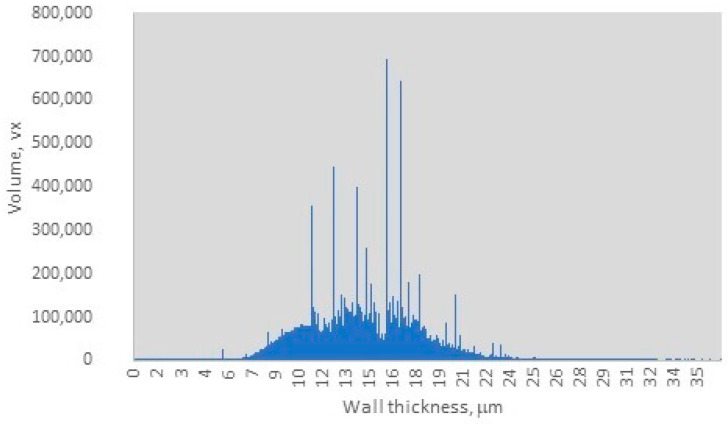
Wall thickness distribution.

The distribution of wall thickness in the charcoal sample, as depicted in Figure 3, indicates that the charcoal wall thickness ranges from 0.05 µm to 25 µm, with a dominant range between 9.5 µm and 18 µm. Within this dominant range, three distinct sharp peaks are observed at 10 µm, 13 µm, and 16 µm, with an average wall thickness of 15 µm and a peak volume of 700,000 voxels. The observed range of wall thickness suggests a moderate degree of heterogeneity in the sample, as evidenced by the relatively narrow distribution of peak values. However, the dominance of specific wall thickness ranges indicates a relative uniformity within the sample, which may influence the final material properties and potential applications. Nisgoski et al. reported that different wood species produce charcoals with distinct wall thickness distributions [33]. Furthermore, they noted that the carbonization process alters the anatomical structures of wood, including vessel diameter and ray width, ultimately impacting the uniformity of wall thickness [33]. This implies that certain samples may exhibit more consistent wall thicknesses due to inherent anatomical characteristics, while others may display greater variability. For instance, by carefully regulating pyrolysis conditions, particularly within the temperature range of 400–600 °C, the structural integrity of cell walls can be maintained while volatiles are released, resulting in thicker walls. In contrast, flash pyrolysis or rapid heating methods often lead to thinner walls and increased heterogeneity due to uneven thermal gradients. Dry wood distillation, on the other hand, provides superior control over the final product quality [33,34,35]. Li et al. reported that archaeological charcoal samples typically exhibit greater cell wall homogenization due to exposure to higher charring temperatures (400–500 °C) compared to experimental charcoals produced at 300 °C. The latter retains more variable wall thicknesses, closely resembling the original wood anatomy. Their findings indicate that the typical wall thickness ranges for different wood types are as follows: dense hardwoods 12–18 µm (broad peaks between at 13–16 µm), softwoods between 8–12 µm (with sharper peaks at 10–12 µm), and Acacia/Terminalia species between 14–20 µm (with a dominant peak at 16 µm) [36]. Additionally, studies by Bhatia, as well as Nguyen and Bhatia, indicated that activated carbons possess pore wall thicknesses ranging between 1.75–3.25 µm [37,38] or consist of 2–3 graphite sheets [36,38]. The dominant wall thickness range observed in this study, with an average wall thickness of 15 µm and a peak volume of 700,000 voxels, Figure 3, supports that the charcoal sample originates from hardwood species, specifically beech wood [36]. Furthermore, these findings suggest that the pyrolysis process likely occurred within a moderate temperature range of 375–425 °C, with the final carbonization stage being conducted at temperatures between 270 °C and 360 °C.

**Figure 4:**
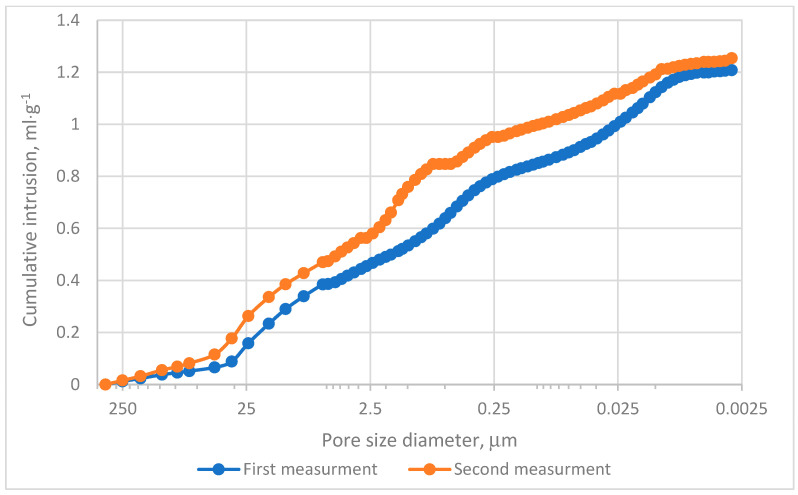
Cumulative intrusion results.

**Figure 5:**
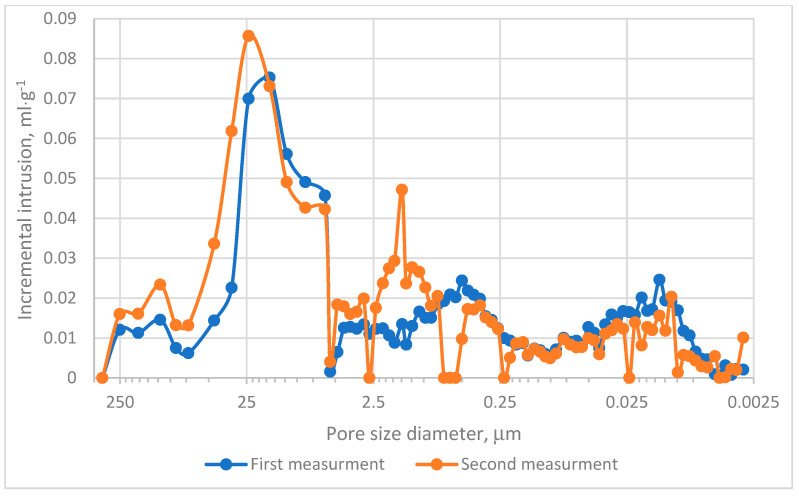
Incremental intrusion results.

The MIP evaluation was conducted in two experimental series. The cumulative intrusion curves demonstrated that pores within the range of 0.0115–0.099 µm predominantly contribute to the mercury intrusion process, as depicted in Figure 4. Analysis of incremental intrusion curves revealed comparable patterns between both series in the range of 5.5–450 µm, Figure 4. The dominant intrusion occurred in macropores within the range of 5–72 µm, Figure 5, with peak intrusion volumes reaching approximately 0.09 mL·g^−1^ in pores measuring depend on series 20–25 µm in diameter, Figure 5. This range aligns with regions of high intrusion rates, as indicated in Figure 4. However, significant differences were noted in the intermediate pore size ranges. Specifically, in Measurement no 2, a secondary intrusion peak of approximately 0.48 mL·g^−1^ was observed within the range of 0.16–5 µm. In contrast, the corresponding intrusion volume in Measurement no. 1 was markedly lower. These discrepancies suggest that the charcoal microstructure under investigation exhibits heterogeneity rather than homogeneity. Furthermore, micropores (pores smaller than 2 nm) exerted minimal influence on the mercury intrusion process. This observation underscores the predominance of larger pores in defining the research material’s intrusion characteristics.

**Figure 6:**
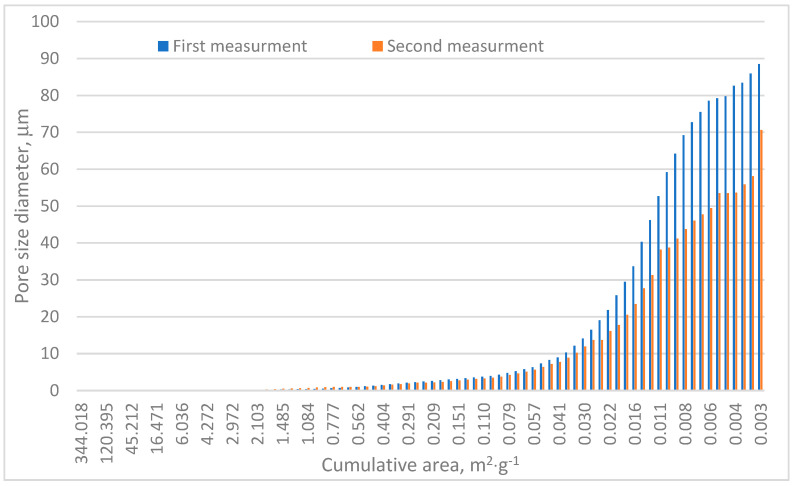
Cumulative area distribution in relation to pore size.

Table 1 and Figure 6 confirm the heterogeneous nature of the analyzed sample. The porosity differences between the two test series reached approximately 4.5%, as shown in Table 1. The total pore area varied significantly, with Measurement no. 1 recording a pore area of 88.5 m^2^·g^−1^ and Measurement no. 2 measuring approximately 70.7 m^2^·g^−1^. Both values notably exceed those reported by Surup et al. and Friesen and Mikula [18,21]. Specifically, Surup et al. identified a total pore surface area of charcoal samples between 11 and 69 m^2^·g^−1^ depending on wood type and temperature, with most values near 11 m^2^·g^−1^ [18]. Similarly, Friesen and Mikula observed surface areas of 14–50 m^2^·g^−1^ in the case of low-rank Canadian coals subjected to temperatures up to 325 °C [21].

The significantly higher surface areas observed in this study can be attributed to the presence of fibrous structures, primarily reported by us [29,39]. These structures may enhance adsorption potential and influence the charcoal’s functional applications. When compared to N_2_ isotherm results from similar charcoal samples [39], which focused on powder forms, the MIP method appears more reliable. The N_2_ isotherms (subjected mostly towards possible application in NO_x_ sorption) yielded surface areas near 20 m^2^·g^−1^, though these values were inconsistent with observed morphological features.

Cumulative area distribution analysis, Figure 6, indicates that the majority of pores fall within the 10–90 µm range. Pores exceeding 10 µm contribute significantly to the cumulative pore surface area and are primarily responsible for mercury adsorption, as shown in Figure 4 and Figure 5. These findings align with pore size distributions reported in other research [39], where most pores measured below 20 µm.

It is worth noting that the high cumulative pore surface observed, Table 1, could also result from the substantial presence of micropores. This observation aligns with the findings of Plotze and Nimz [40]. However, micropores have limited accessibility, as confirmed by CT scans and the lack of pore size distribution data in the CT scan module, which additionally enhanced the application of MIP. Studies on coal employing MIP and SEM have demonstrated that multiscale pore structures are significantly influenced by coal rank and fractal dimensions [41,42]. High-rank coals predominantly feature micropores, which govern their adsorption properties, whereas lower-rank coals exhibit a greater proportion of mesopores and macropores [42]. Charcoal exhibits notably higher porosity (up to 62%) compared to typical coal porosity values (approximately 30–50%) [42]. However, MIP studies on wood-based oriented strand board (OSB) panels indicate lower porosity values (approximately 40–50%) relative to charcoal [43]. As was mentioned, tomographic imaging of charcoal revealed structural alignment along wood fibers, similar to OSB, but with greater heterogeneity, which is visible in Table 1 and Figure 6, due to the presence of seasonal growth rings and thinner cell walls in knot regions. These structural variations contribute to localized increases in pore size, which may enhance adsorption efficiency in specific regions.

Considering the results obtained from CT and MIP analyses, it can be concluded that the observed MIP deviations arise from both structural heterogeneity and methodological limitations. Firstly, the presence of larger pores and thinner material walls in knot regions, as observed in CT scans, Figure 2c,f, created localized high-porosity zones, contributing to variability in MIP measurements [44]. Secondly, seasonal variations in wood structure lead to differences in pore size and wall thickness, which could have influenced the mercury intrusion pathways. Thirdly, the presence of aligned fibers and cross-sectional variations, as evident in Figure 2a–f, resulted in uneven pore connectivity, which MIP struggles to resolve due to its reliance on pressure-driven intrusion (enhances mercury intrusion into aligned pores but masks micropores) [45]. Additionally, factors such as the ink-bottle effect, micropore accessibility, and material compressibility may further contribute to MIP deviations between the two test series. In complex pore geometries (e.g., narrow necks leading to larger voids), MIP tends to overestimate pore sizes, as mercury intrudes through the narrowest constriction while filling larger chambers [45,46]. Moreover, although micropores dominate the surface area, MIP fails to resolve them effectively due to limitations in pressure ranges or pore throat accessibility [46,47]. Furthermore, coal and charcoal matrices exhibit a compressibility of approximately 1.342·10^−4^ MPa^−1^ in raw coal, which can distort pore volume calculations under high-pressure conditions [45]. However, despite these methodological influences, the dominant factor contributing to MIP deviation remains the inherent structural heterogeneity of the sample.

**Figure 7:**
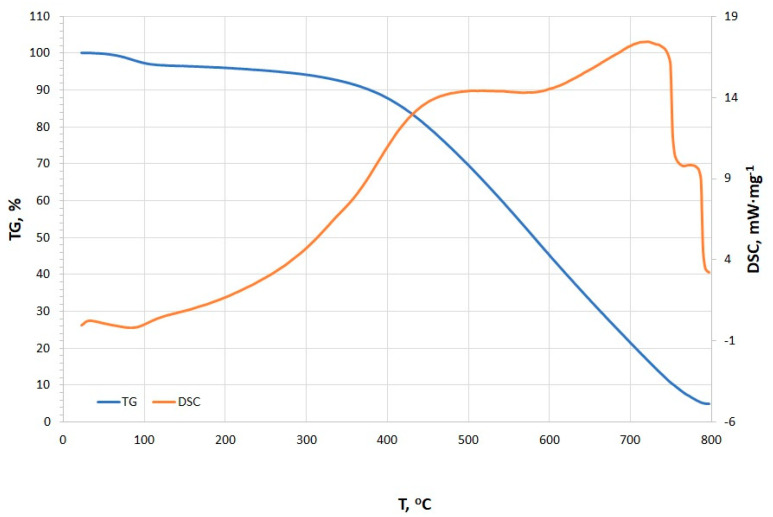
TG/DSC curves of microstructure charcoal.

The TG and DSC curves presented in Figure 7 reveal distinct thermal events occurring at various temperature points, including dehydration, adsorption/absorption and oxidation, pyrolysis, and burnout (stabilization) [48]. Initially, a minor endothermic peak is observed at 35.4 °C (0.242 mW·mg^−1^), which is likely associated with the dehydration process, corresponding to the evaporation of surface-bound water molecules [48,49,50]. With increasing temperature, an exothermic peak is detected at 89.6 °C (−0.138 mW·mg^−1^), possibly indicating adsorption or desorption of water vapor and other volatile compounds [48,50]. The presence of this peak is unusual for typical charcoal. Liang and Cameron suggested that such low-temperature exotherms are rare in pure charcoals but observed in impregnated carbons (e.g., active surface charcoal with Cu) [51]. This suggests a unique surface chemistry which could also be a result of the fibrous structures that were observed under the SEM. The onset of pyrolysis is identified between 488.2 °C and 588.5 °C, with a peak intensity of 14.4 mW·mg^−1^. The relatively consistent energy demand within this temperature range suggests the decomposition of similar organic compounds. At this stage, the organic matter within the charcoal undergoes thermal degradation, leading to the release of volatile gases and the formation of solid residues. However, overlapping endothermic peaks signify pyrolysis of organic components (e.g., lignin-derived structures). These align with fresh charcoals but are slightly shifted compared to aged chars, which often show higher peak temperatures (>500 °C) [52]. The DSC peak at 728.9 °C marks the advanced phase of pyrolysis, potentially coupled with the combustion of carbonaceous residues [48,50]. The observed peak is much higher in comparison to the beech wood charcoal. The stabilization phase, occurring in the temperature range of 763.9–800.0 °C, is characterized by a gradual reduction in heat flow intensity, indicating the completion of pyrolysis reactions and the transition of the remaining material into a thermally stable char phase [48,50]. This interpretation is supported by the progressive decline in peak intensity. Moreover, the observed burnout phase is similar to mineral-rich charcoal with residual ash oxidation [52].

The TG/DSC results are consistent with our previous studies [29], where the primary difference lies in the grain size of both samples. Furthermore, considering both the sample origin and thermal behavior, it can be inferred that the investigated samples will exhibit similar XPS characteristics, as reported in [29].

## 4. Conclusions

Detailed analysis of a microstructured charcoal sample with a fibrous carbon structure was conducted by SEM, CT, MIP, and TG/DSC.

Obtained CT and MIP results confirmed the heterogeneous character of the studied sample. The observed differences between two measurement series using MIP indicate variations in the pore size distribution and potential connectivity of the tested charcoal sample. MIP Measurement no. 1, characterized by a higher pore area and overall porosity, likely possesses a more interconnected microporous network, which enhances its adsorption efficiency for smaller molecules. In contrast, the larger average pore diameter observed in MIP Measurement no. 2 suggests a predominance of mesopores, potentially facilitating the adsorption of larger molecular species. These differences obtained from two research series of the same MC sample can be mostly explained by its structural heterogeneity. Further evaluation can be directed to the examination of the pore connectivity.

The hierarchical pore structure of charcoal, encompassing macropores, mesopores, and micropores, provides adaptability for the adsorption of molecules of varying dimensions. Additionally, the fibrous structures observed at higher magnifications of SEM further increase the available surface area of the sample, rendering charcoal a promising material for applications requiring high adsorption efficiency, such as water purification and gas storage. Moreover, the uniform wall thickness should influence the mechanical stability of the charcoal sample and predictable porosity, making it suitable for applications such as filtration or as a potential precursor for activated carbon.

The total pore area determined via MIP, ranging from 70.7 to 88.5 m^2^·g^−1^, corroborates that the previously conducted pore surface assessment using nitrogen (N_2_) adsorption was insufficient. This suggests that in cases where a substantial presence of microporous structures is likely, adsorption isotherm-based methods may underestimate the actual surface area. Consequently, a comprehensive pore characterization necessitates the combined application of MIP and N_2_ adsorption to achieve a more accurate and reliable evaluation of the material’s porosity.

## Figures and Tables

**Table 1:** MIP results of microstructure charcoal sample.

Parameter	Measurement No. 1	Measurement No. 2
Total intrusion volume, mL·g^−1^	1.2078	1.2543
Total pore area, m^2^·g^−1^	88.496	70.662
Average pore diameter, μm	0.0546	0.0710
Porosity, %	62.4143	58.0625

## Data Availability

The original contributions presented in this study are included in the article. Further inquiries can be directed to the corresponding author.
